# Programming Gels Over a Wide pH Range Using Multicomponent Systems

**DOI:** 10.1002/anie.202101247

**Published:** 2021-03-24

**Authors:** Santanu Panja, Bart Dietrich, Olga Shebanova, Andrew J. Smith, Dave J. Adams

**Affiliations:** ^1^ School of Chemistry University of Glasgow Glasgow G12 8QQ UK; ^2^ Diamond Light Source Ltd. Diamond House Harwell Science and Innovation Campus Didcot Oxfordshire OX11 0DE UK

**Keywords:** ambidextrous phase behavior, autonomous programming, cooperative hydrogen bonding, multicomponent hydrogels, pH responsiveness

## Abstract

Multicomponent hydrogels offer a tremendous opportunity for preparing useful and exciting materials that cannot be accessed using a single component. Here, we describe an unusual multi‐component low‐molecular weight gelling system that exhibits pH‐responsive behavior involving cooperative hydrogen bonding between the components, allowing it to maintain a gel phase across a wide pH range. Unlike traditional acid‐triggered gels, our system undergoes a change in the underlying molecular packing and maintains the β‐sheet structure both at acidic and basic pH. We further establish that autonomous programming between these two gel states is possible by an enzymatic reaction which allows us to prepare gels with improved mechanical properties.

## Graphical abstract

We show that a multicomponent supramolecular gel with two opposite ionizable pendant groups on different components can exhibit unusual phase behavior involving cooperative hydrogen bonding between the components, allowing it to maintain a gel phase across a wide pH range. Autonomous programming between these two gel states is possible by an enzymatic reaction which allows the preparation of gels with improved mechanical properties.

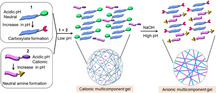

Supramolecular gels formed by the self‐assembly of amphiphiles have huge potential in many areas including biomaterials and optoelectronics.^[1]^ To bring about gelation, usually a trigger such as pH, temperature, light, or salt is applied to the solution or suspension of the amphiphile to significantly reduce the solubility of the molecules. Consequently, a balance between the hydrophobicity and hydrophilicity of the amphiphile drives fibre formation and gelation.^[2]^ When a counter trigger is applied that significantly perturbs the hydrophobic/hydrophilic balance of the amphiphile, supramolecular gels typically return to the non‐gelling solution (or sol) state.

In comparison, a class of materials (unfortunately) often named “schizophrenic” represent a special class of self‐assembled system where the building blocks transform themselves either into a hydrophobic or hydrophilic unit in response to a change in solution pH, temperature, or ionic strength, and maintain the assembled structures on perturbation.^[3]^ Since the first reports of this type of micellization in 1998,^[3a]^ amphiphiles with this behavior have been used to construct stimuli‐tunable switchable assemblies for nanoreactors, drug delivery and controlled release of encapsulated materials.[^3b^, ^3d^, ^4^] Most of these amphiphiles are polymeric in nature, where the backbone contains two types of functional groups. The individual functional units can be independently tuned to become either hydrophilic or hydrophobic. The phase behavior of such diblock copolymers arises from the response of the individual functional blocks during perturbation.[^3a^, ^3b^, ^5^]

Incorporation of such ambidextrous phase behavior into supramolecular gel systems also has the potential to be an effective strategy to synthesize pH switchable gels and adaptive materials but has yet to be reported. Here, we devise a multicomponent hydrogel system that exhibits pH‐responsive behavior involving cooperative hydrogen bonding between the components. Multicomponent gels are typically comprised of two or more independent gelators^[6]^ and offer opportunity to prepare useful materials that cannot be obtained using a single component.^[7]^ pH triggered multicomponent gels are common.[^6a^, ^7c^, ^8^] However, the challenge here is that such gels are mostly stable at acidic pH whilst at a pH above the apparent p*K*_a_ of the gelators, formation of corresponding carboxylate ions leads to sol formation. Hence, these systems do not maintain their assembled structures on pH perturbation. Here, we show a new approach, designing a multicomponent hydrogel comprising of two opposite ionizable pendant group on the different gelator backbones can exhibit a gel‐to‐gel transition on reversal of the pH (Figure 1). We also establish that autonomous programming between these two pH‐dependent gel states is possible by introducing the autocatalytic urease‐urea reaction to access gels with more controlled properties, and critically maintain a gel phase across a wide pH range.

**Figure 1:**
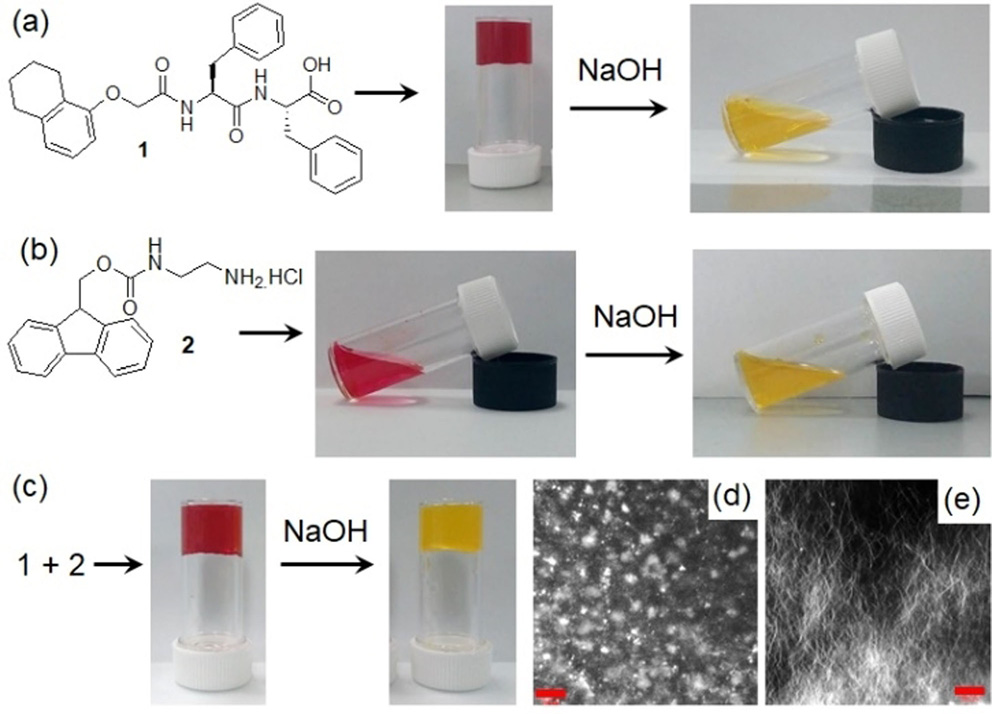
a–c) Photographs showing the phase transformations of **1** and **2** in absence and presence of NaOH. Confocal fluorescence microscopy images (scale bars=20 μm) of the multicomponent gel of **1** and **2** obtained in d) absence and e) presence of NaOH. In all cases, initial concentrations of **1** and **2** are 2 mg mL^−1^ (2 mg mL^−1^ of each in the mixed gel), concentration of NaOH is 0.01 M, solvent is DMSO/H_2_O (20:80, v/v). Methyl red (0.05 mg mL^−1^) is used to stain the gels (and sols).

Initially, we prepared the multicomponent gel by mixing compounds **1** and **2** in DMSO/H_2_O (20/80, v/v) at a concentration of 2 mg mL^−1^ (Figure 1). Both **1** and **2** contain pH‐dependent ionizable functional groups and the solubility of the molecules can be controlled by the degree of protonation or deprotonation of these groups.

The apparent p*K*_a_ of **1** and **2** is 6.4^[9]^ and 8.6 (Figure S5, Supporting Information) respectively in DMSO/H_2_O (20/80, v/v). **1** alone forms a gel with a pH of around 4.3 when water is added to a solution of **1** in DMSO (Figure 1 a).^[9]^ The small angle X‐ray scattering (SAXS) data for this gel fit well to a cylinder model combined with a power law to take into account the excess scattering at low Q (Figure 2 a). The cylinders have a radius of 3.5 nm, and a length greater than that accessible by this technique. Compound **2** alone remains in solution under similar conditions (Figure 1 b) with a pH of 5.3. The SAXS data for this sample fit to a power law only with low scattering intensity, suggesting a lack of significant aggregation (Figure 2 a). Combining **1** and **2** results in a gel being formed, with the pH of the multicomponent gel being pH 3.3 (Figure 1 c). The SAXS data for this mixture fit to a cylinder and power law model as for **1** alone but suggest that the radius is slightly larger (4.5 nm; Figure 2 a).

**Figure 2:**
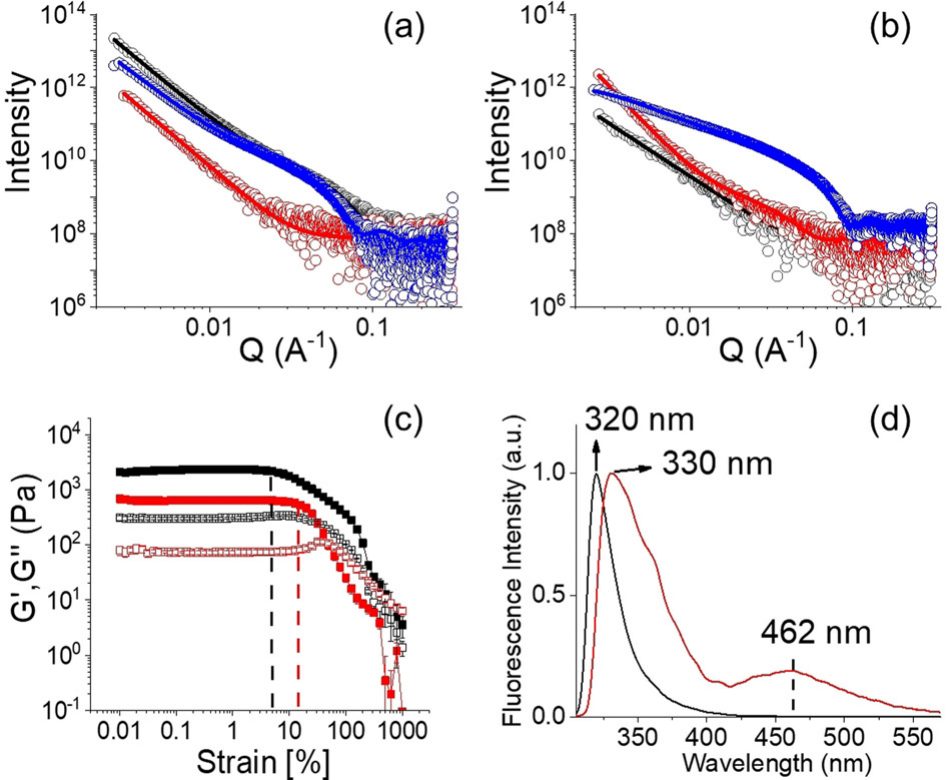
a) SAXS data and fits for systems at low pH. b) SAXS data and fits for systems at high pH. For (a) and (b), the black data are for **1** alone, the red data for **2** alone and the blue data for the mixture of **1** and **2**. The open symbols show the data and the solid lines the fits to the data as discussed in the text. c) Strain sweep experiments and d) normalized emission spectra of the hydrogels of (**1**+**2**) obtained at pH 3.3 (black) and pH 10.2 (red). For (c), the dotted vertical lines represent the maximum strain bearing capacity of the hydrogels of (**1**+**2**) obtained at pH 3.3 (black) and pH 10.2 (red). In all cases, initial concentrations of **1** and **2** are 2 mg mL^−1^, concentration of NaOH is 0.01 M, solvent is DMSO/H_2_O (20:80, v/v). For (c), the closed symbols represent G′, the open symbols G′′.

The multicomponent gel and the gel of **1** also have some differences in their microstructure as shown by confocal microscopy imaging (Figure 1 d and Figure S6). The multicomponent gel contains a higher density of spherulitic nucleation centers compared to the hydrogel formed from **1** alone. We hypothesise that the reason for increasing density of spherulitic nucleation centres in the multicomponent gel can be ascribed to the salt effect arises due to the presence of hydrochloride salt **2** affecting the structuring of the peptide network involving the Hofmeister effect. Dissolving a salt in water significantly change the water structure and dynamics^[10]^ which leads to differences in hydration of **1**.^[11]^ Consequently, there was a change in the nucleation centre followed by the growth of fibres.^[12]^ However, no significant change in the rheological moduli (the storage and loss moduli, G′ and G′′) of the gels was noted (Figure S6 and S7). Hence, in the multicomponent gel, while compound **1** acts as a gelator, compound **2** behaves as a non‐gelling additive that influences the assembly of **1** and so the gel properties.^[6a]^ At basic pH, while the deprotonated forms of both **1** and **2** behave as non‐gelling components individually, a stable gel was obtained from their mixture at a pH of 10.2 (Figure 1 and Figure S8). At high pH, the carboxylic acids of **1** are ionized and so are unable to sustain the gel network,^[9]^ but deprotonation of the ammonium group of **2** generates neutral amine derivative and so can trigger self‐assembly by maintaining the overall hydrophobic/hydrophilic balance involved in co‐assembly (Figure 3). Hence, the mixture of **1** and **2** forms gels at both high and low pH. Underlying this behavior is a change in gel network from spherulitic structures to long fibers on changing from acidic to basic pH (Figure 1 d, 1e). The SAXS data for **1** alone at high pH are of low intensity and fit to a power law only, suggesting a lack of significant aggregation (Figure 2 b). **2** alone at high pH forms cylindrical structures (again a power law is needed to take into account the scattering at low Q) (Figure 2 b). However, the data for the mixture fit well to a flexible cylinder model and clearly differ from that of either component (Figure 2 b). There were also substantial differences in the mechanical properties of the gels. While the low pH gel showed higher stiffness (G′), the strain bearing capacity (the strain at which the gel breaks) of the gel increased remarkably at high pH (Figure 2 c).

**Figure 3:**
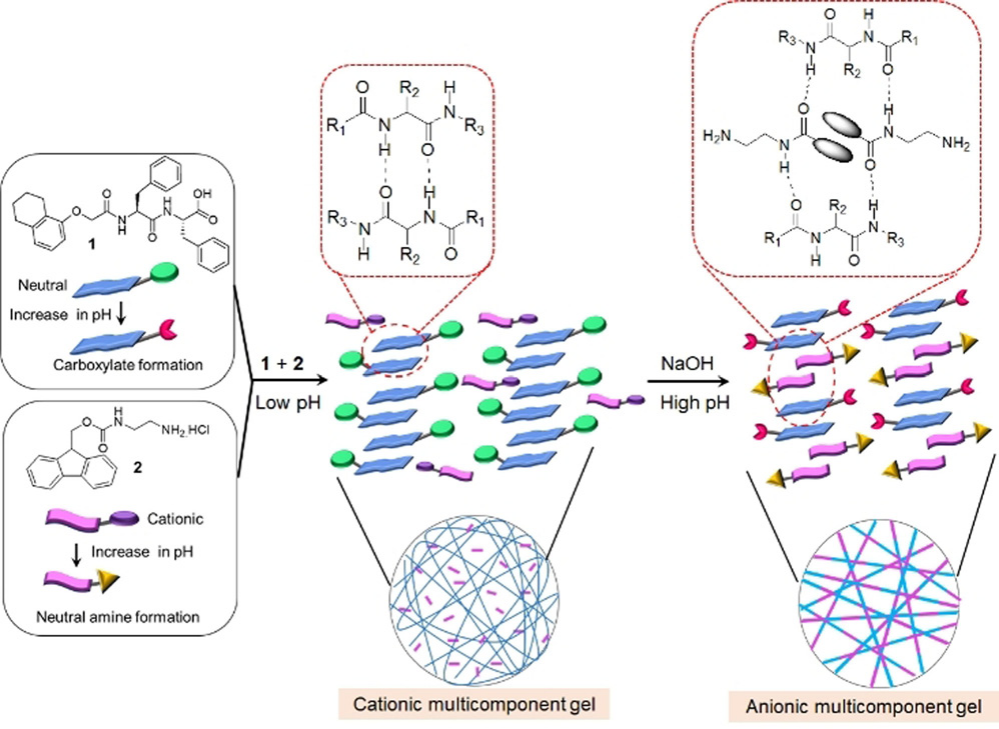
Probable modes of aggregation of **1** and **2** in the multicomponent gel at different pH.

To understand the mechanism of gelation under different conditions, FTIR spectra of **1** and **2** were collected (Figure S9 and S10). In the presence of **1** alone, the stretching signals at 1687 cm^−1^ and 1648 cm^−1^ in the gel state at low pH suggest formation of antiparallel β‐sheet‐like structures through intermolecular hydrogen bonding involving the amide carbonyls.^[13]^ For the multicomponent gel at low pH, while the peak at 1647 cm^−1^ remains unaffected, the shoulder at 1687 cm^−1^ becomes stronger due to overlapping with the carbamate carbonyl of **2** and shifted to 1681 cm^−1^. Interestingly, the carboxylic carbonyl of **1** which appeared at 1723 cm^−1^ in its gel state, became too broad to distinguish in the multicomponent gel. The signals for the amide ‐NHs and carboxylic‐OH of **1** merged with the ‐NH stretching of **2** and appeared at 3280 cm^−1^ as a sharp signal in the multicomponent gel. In presence of base at high pH, while the peak at 1648 cm^−1^ remains unaffected, the stretching signal at 3280 cm^−1^ became broad. At high pH, a new peak appeared at 1634 cm^−1^ signifying involvement of **2** in retaining the antiparallel β‐sheet structures.^[14]^ Furthermore, the peak at 1681 cm^−1^ for the carbamate carbonyl of **2** moved higher by 3 cm^−1^ in presence of base indicating a different type of molecular packing at high pH; this was further confirmed by UV‐vis and fluorescence studies. In fluorescence, the solution of **2** at low pH exhibits monomer emission at 318 nm (Figure S11). In the multicomponent gel, the emission wavelength of **2** merged with the emission of **1** and appeared at 320 nm but experiences a 10 nm red shift with reduced intensity on changing the pH from acidic to basic (Figure S11, S12, Figure 2 d). Interestingly, the high‐pH gel exhibited excimer emission in the region 425–525 nm characteristic of overlapping of the fluorenyl groups.^[15]^ This was confirmed by recording the fluorescence spectra of the individual components where the emission in the region 425–525 nm was only observed for **2** at high pH (Figure S11). A dilution in the concentration of **2** at high pH diminished the band in the region 425–525 nm and further corroborates the excimer formation due to overlapping of the Fmoc‐moieties (Figure S13).[^15b^, ^15d^, ^16^] We also recorded emission spectra of the mixture of **1** and **2** at different compositions of the gelators. At a fixed NaOH concentration, an increase in relative concentration of **2** with respect to **1** resulted in an increase in intensity of the excimer emission again endorses better overlapping of the Fmoc‐groups at higher concentration of **2** (Figure S14).[^15c^, ^17^] On the other hand, addition of base resulted in a red shift of around 2 nm in the absorption spectra of the gel (Figure S15). These results demonstrate a difference in underlying molecular packing of the gels formed at different pH.

We next incorporate the urease‐urea reaction to pre‐program the transition between two pH‐dependent states of our system. Hydrolysis of urea by urease produces ammonia over time which in turn increases the pH of the medium.^[18]^ This reaction is widely used to achieve precise control over the rate of pH change and thereby control material properties.[^9^, ^19^] No substantive change in the rate of pH change by the enzyme was observed in DMSO/H_2_O (20/80, v/v) compared to normal water (Figure S16). When we mixed **1** and **2** in presence of urea and urease in DMSO/H_2_O, initially a gel was obtained at pH 3.3, with G′ significantly greater than G′′ which shows that a gel is formed (Figure 4 a).

**Figure 4:**
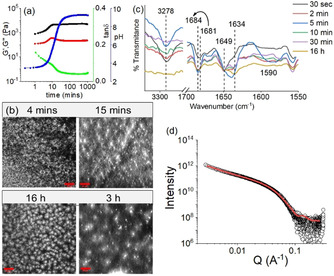
a) Variation of pH (blue), G′ (black), G′′ (red) and tanδ (green) with time for the mixture of **1** and **2** involving urea–urease reaction. b) Time dependent confocal microscopy images and c) partial FTIR spectra of the mixture of **1** and **2** involving urea‐urease reaction. Scale bars=20 μm. d) SAXS data (circles) and fits (red line) for the mixture of **1** and **2** at high pH obtained from the enzymatic reaction. In all cases, initial concentrations of **1** and **2** are 2 mg mL^−1^, [urease]=0.5 mg mL^−1^, [urea]=0.1 M, solvent is DMSO/H_2_O (20/80, v/v).

The urease‐urea reaction is itself pH‐dependent, and, below pH 4, the activity of urease is low. Hence, at the early times, the pH change was slow. However, after pH 4 was reached, the rate of the production of ammonia increases, which results in a sharp increase in the pH of the medium. The pH‐time profile (Figure 4 a) shows a sigmoidal curve where the pH reached a plateau of 9.2 after 2 hours. The rheological moduli also evolve with time, but critically show that a gel phase is maintained across the whole pH range from below 4 to above 9. The high pH gel exhibits considerably higher values of both G′ and G′′ than the initially formed gel (Figure 4 a, Figure S17, S18). During this pH evolution, no obvious phase transformation was noticed; however, a change in gel microstructure was apparent in confocal microscopic studies which revealed a decrease in density of the spherulitic aggregates at high pH (Figure 4 b). Time variable FTIR spectra showed that with increase in pH, the intensity of the peak at 3278 cm^−1^ progressively decreased (Figure 4 c). Simultaneously, a broad signal appearing at 1590 cm^−1^ after 5 mins corresponding to the formation of carboxylate ion gradually disappeared with further time.^[20]^ The emergence of a new peak at 1634 cm^−1^, ascribed to the presence of antiparallel β‐sheets, validates the existence of a different type of hydrogen bonded network between **1** and **2** at different pH (Figure 3). Furthermore, 4 nm and 17 nm red shifts in the absorption and emission spectra, respectively of the gel again show that there is a change in the molecular packing of the underlying structures as the pH increases (Figure S19). Interestingly, the self‐regulating approach here leads to a material which exhibits >3 times higher stiffness with a different microstructure than the gel obtained directly at high pH (Figure 1 e, Figure 4 b, Table S3). In agreement with these differences, the SAXS data for the mixture at high pH induced by the enzymatic pH change are different to those obtained by using NaOH (Figure 2 b, Figure 4 d, Table S2); the data can best be fitted in this case to a flexible elliptical cylinder, showing that the differences in the rheology can be directly linked to differences in underlying self‐assembled structures.

The transition between the different states can be controlled by adjusting the rate of pH change. As ammonia production depends on the concentration of both urease and urea, a decrease in any of their concentrations (keeping other parameters fixed) substantially delays the pH increase (Figure 5). However, no significant difference in the final pH was observed. Rheology shows that instead of a sharp change in G′ and G′′ as observed when the pH change is fast, a slow but steady increase in rheological moduli with time was noted with slower pH changes (Figure 5 a, S20). Interestingly, when the rate of pH change is slow, a decrease in G′ and G′′ was noticed in the high pH region. We presume that when the pH change is fast, kinetically trapped structures are formed which prevent further structural rearrangements with time. At a slow rate of pH change, the hydrogen bonded co‐assembled structures reorganize again at high pH to attain lower‐energy structures which exhibit a slightly higher intensity of the excimer peaks in fluorescence (Figure S21).^[21]^ Evaluation of tanδ (G′′/ G′) shows that the solid‐like nature of the gels persists throughout the high‐pH regime. A 40 % reduction in either urease or urea concentration leads to a material that exhibits ≈50 % reduction in gel stiffness (Figure S22, Table S4).

**Figure 5:**
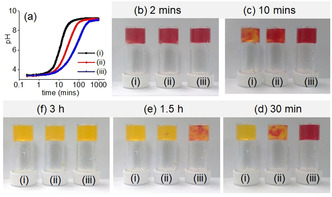
a) Variation of pH with time along with b–f) associated color change for the mixtures of **1** and **2** involving urea‐urease reaction. Initial conditions: (i) [urease]=0.5 mg mL^−1^, [urea]=0.1 M; (ii) [urease]=0.5 mg mL^−1^, [urea]=0.06 M; (iii) [urease]=0.3 mg mL^−1^, [urea]=0.1 M. In all cases, initial concentrations of **1** and **2** are 2 mg mL^−1^, solvent is DMSO/H_2_O (20:80, v/v). For (b–f), methyl red (0.05 mg mL^−1^) is used to stain the gels.

Cross‐linking of the self‐assembled aggregates formed by **1** at high pH is possible by divalent cations.^[21]^ With this in mind, we incorporated Ca^2+^ into our system. An increased concentration of Ca^2+^ resulted in an increase in the rate of pH change and thereby accelerated increase of both G′ and G′′ (Figure 4 a, S23). Interestingly, the rheological moduli of the final gels progressively increase with an increase in Ca^2+^ ion concentration (Figure S24, Table S5). Following the process using UV‐vis spectroscopy showed that there was an increase in intensity of the shoulder peak at 278 nm under both conditions although no major change in the emission properties of the gels was noticed (Figure S25).

In comparison, an increase in the concentration of either **1** or **2** (keeping other parameters fixed) resulted in a delay in the rate of pH increase and thereby influenced rheological changes (Figure S26). In these cases, the stiffness of the final gels increases considerably (Figure S27, Table S6).We presume that at high pH, while compound **1** forms a micellar dispersion, compound **2** exists in a relatively hydrophobic amine structure. Both these forms aid to stabilize the hydrogen bonded network and thereby increase the stiffness of the materials. The shoulder peak at 278 nm intensified remarkably and became broad in both cases (Figure S28). While an increased concentration of **1** led to a larger red shift in monomer emission (21 nm) of the final gel, the relative intensity of the excimer peaks dramatically increased with an increase in concentration of **2** (Figure S28). These results show that, while the micellar dispersion of **1** tends to destroy aromatic stacking, an increased concentration of **2** extends the overlapping of the fluorenyl groups.^[15c]^

Correlation of pH‐time profiles further indicates that, in spite of significant differences in the rate of pH change, no substantial change in the pH of the final gels was noticed. There was no considerable difference in the microstructure of the final gels either.

In conclusion, we have shown that a multicomponent supramolecular gel with two opposite ionizable pendant groups on different components can exhibit unusual phase behavior and allows access to two different gel states both at acidic and basic pH. We also establish that autonomous programming between these two pH‐dependent gel states is possible by incorporating the autocatalytic urease‐urea reaction which allows us to achieve an unprecedented degree of control over the final mechanical properties of the gels. Unlike traditional pH‐triggered multicomponent systems where a change in pH collapses the gel network, our system undergoes a change in molecular packing and maintains the gel network structure across a wide pH window involving cooperative hydrogen bonding between the components. The results further emphasize the necessity of engineering autonomous behavior in synthesizing next generation switchable assemblies. This methodology extends the useful range of such materials, critically incorporating physiological pH.

## Conflict of interest

The authors declare no conflict of interest.

## Supporting information

As a service to our authors and readers, this journal provides supporting information supplied by the authors. Such materials are peer reviewed and may be re‐organized for online delivery, but are not copy‐edited or typeset. Technical support issues arising from supporting information (other than missing files) should be addressed to the authors.

Supplementary
