# Supplementary material for: Programming Gels Over a Wide pH Range Using Multicomponent Systems
Source: Angew Chem Int Ed Engl. 2021 Mar 24;60(18):9973–7. doi: 10.1002/anie.202101247 (PMC8252051; doi:10.1002/anie.202101247)
Supplement: Supplementary file 1 — Supplementary [file ANIE-60-9973-s001.pdf]

Angewandte Chemie
Eine Zeitschrift der Gesellschaft Deutscher Chemiker

Supporting Information

# Programming Gels Over a Wide pH Range Using Multicomponent Systems

*Santanu Panja, Bart Dietrich, Olga Shebanova, Andrew J. Smith, and Dave J. Adams**

anie_202101247_sm_miscellaneous_information.pdf

# Programming gels over a wide pH range using multicomponent systems

Santanu Panja,[a] Bart Dietrich,[a] Olga Shebanova,[b] Andrew J. Smith[b] and Dave J. Adams[a]*

a School of Chemistry, University of Glasgow, Glasgow, G12 8QQ, U.K.

b Diamond Light Source Ltd., Diamond House, Harwell Science and Innovation Campus, Didcot, Oxfordshire OX11 0DE, U.K.

*Email: dave.adams@glasgow.ac.uk*

## Supporting Information

**Table of Contents**


Experimental Details S3

Supplementary Figures S7

References S17

**Experimental details**

**Materials:** Compound **1** was synthesised as described previously.[1] Urease (J61455 Urease, Jack Beans, minimum 45.0 units/mg solid) and urea (ultrapure 99%) were obtained from Alfa Aesar. Compound **2** was purchased from Sigma Aldrich and used as received. $CaCl_2$ (granular) was obtained from Fisher Scientific. Deionised water was used throughout all experiments.

**Preparation of solutions:** Stock solutions of **1** were prepared in DMSO at concentrations of 10 mg/mL and 15 mg/mL by stirring. A stock solution of **2** was prepared at a concentration of 5 mg/mL in water by stirring. Stock solutions of urea, urease and $CaCl_2$ were prepared in $H_2O$ in concentrations of 4 M, 5 mg/mL and 1 M, respectively. The enzyme, urea and $CaCl_2$ were highly soluble in $H_2O$ and therefore did not require stirring. The enzyme concentration in the stock solution was determined from the mass (in mg) dissolved in a known volume of $H_2O$. Solutions of all the components were prepared freshly before each experiment. Stock solution of NaOH was prepared at a concentration of 0.1 M in $H_2O$.

**Preparation of gels:** Hydrogels of **1** were prepared by adding 1.60 mL of $H_2O$ to 0.4 mL of the DMSO solution of **1** (10 mg/mL) in one aliquot. Therefore, the ratio of DMSO and water was 20:80 and the concentration of **1** was 2 mg/mL.
The multicomponent hydrogels were prepared from a mixture of **1** and **2** in DMSO/$H_2O$ (20/80, v/v) under different conditions. To prepare the gels at pH 3.3, 0.40 mL of the solution of **1** (10 mg/mL) was transferred into a 7 mL Sterilin vial. To this solution, a mixture of 0.8 mL of $H_2O$ and 0.8 mL of solution of **2** was added in one aliquot. To prepare the multicomponent gels at pH 10.2, a mixture of 0.6 mL of $H_2O$ and 0.8 mL of solution of **2** was transferred to the vial containing a mixture of 0.4 mL of **1** (10 mg/mL) and 0.2 mL of NaOH. Therefore, in the respective gels, initial concentrations of **1** and **2** were 2 mg/mL and concentration of NaOH was 0.01 M. In both cases, the ratio of DMSO and water was 20:80.
To prepare gels involving the enzymatic reaction, a common procedure was followed. For these experiments, a mixture of **2** and urease was prepared by diluting urease solution with water followed by addition of **2**. The mixture was then immediately transferred to the solution of **1** or a mixture of **1** and urea. Initially, the multicomponent gels of **1** and **2** were prepared at low pH in presence of urease but in absence of urea by diluting 0.4 mL of DMSO solution of **1** (10 mg/mL) with a mixture of 0.6 mL of $H_2O$, 0.2 mL of solution of urease and 0.8 mL of solution of **2**. Therefore, the concentrations of **1** and **2** were 2 mg/mL and concentration of urease was 0.5 mg/mL.
To prepare the gels in presence of both urease and urea, a mixture of $H_2O$ (0.55 mL, 0.57 mL or 0.63 mL, as required), 0.8 mL of solution of **2** and urease (0.2 mL or 0.12 mL, as required) was transferred to the vial containing 0.40 mL of **1** (10 mg/mL) and urea (50 µL or 30 µL, as required). Therefore, in the respective gels, initial concentrations of **1** and **2** were 2 mg/mL, concentration of urease was 0.5 mg/mL or 0.3 mg/mL (as required) and initial concentration of urea was 0.1 M or 0.06 M (as required). In a different set, a mixture of $H_2O$ (0.55 mL or 0.15 mL, as required), solution of **2** (0.8 mL or 1.2 mL, as required) and urease (0.2 mL) was added to a mixture of 0.40 mL of **1** (15 mg/mL or 10 mg/mL, as required) and urea (50 µL) such that the initial concentration of **1** was 3 or 2 mg/mL (as required), initial concentration of **2** was 2 or 3 mg/mL (as required), concentration of urease was 0.5 mg/mL and initial concentration of urea was 0.1 M.
For the enzymatic reactions in presence of $Ca^{2+}$, a mixture of 0.55 mL of $H_2O$, 0.2 mL of solution of urease and 0.8 mL of solution of **2** was added to a mixture of 0.4 mL DMSO solution of **1** (10 mg/mL), urea (50 µL) and $CaCl_2$ (either 4 µL or 12 µL). Hence, the initial concentrations of **1** and **2** were 2 mg/mL, initial concentration of urea was 0.01 M, concentration of urease was 0.5 mg/mL and concentration of $CaCl_2$ was either 0.002 M or 0.006 M.
In all cases, the gels were prepared in a volume of 2 mL in which the ratio of DMSO and $H_2O$ was maintained at 20:80. All samples were left overnight before measurements were carried out.

**pH measurements:** A FC200 pH probe from HANNA instruments with a 6 mm x 10 mm conical tip was used for pH measurements. The stated accuracy of the pH measurements is ±0.1. For the urea-urease reaction involving compounds **1** and **2**, the reaction mixtures were prepared as described above at a 2 mL volume in a 7 mL Sterilin vial and the pH change was monitored with time. The temperature was maintained at 25 °C during the measurement by using a circulating water bath.
p$K_a$ determination was carried out by recording the pH values after each addition of HCl (0.1M) to the individual solution of **1** and **2** (concentration is 2 mg/mL) containing 1 molar equivalents of NaOH (0.1 M) in 20% DMSO in $H_2O$. During the titrations, to prevent any gel formation, the solutions were stirred continuously. The experimental temperature was 25 °C.

**Rheological measurements:** All rheological measurements were undertaken on an Anton Paar Physica MCR 301 rheometer at 25 °C. Strain, frequency and time sweeps were performed using a vane and cup geometry. Strain sweeps were performed at 10 rad/s from 0.01 % to 1000 % strain. Frequency sweeps were carried out from 1 rad/s to 100 rad/s at 0.5 % strain. All gels were left ~16 hours before being measured. Time sweeps were performed at an angular frequency of 10 rad/s and with a strain of 0.5%. For all experiments, gels were prepared as mentioned earlier in 2 mL volume in a 7 mL Sterilin vials.

**Confocal microscopy:** A Zeiss LSM710 confocal microscope (Zeiss, Gottingen, Germany) with an LD EC Epiplan NEUFLUAR 50X, 0.55 DIC (Carl Zeiss, White Plains, NY, USA) objective was used for imaging. Samples were prepared as mentioned earlier containing Nile blue (2 μL/mL of a 0.1 wt % solution) in CELLview culture dishes (35 mm diameter) and were excited at 633 nm using a He-Ne laser. Images were captured using Carl Zeiss ZEN 2011 v7.0.3.286 software.

**UV-Vis measurements:** Absorption spectra of **1** and **2** under different conditions were recorded on an Agilent Technologies Cary 60 UV-Vis spectrophotometer using a 0.01 mm path length quartz cuvette. All gel samples were prepared in Sterilin vials using the same methodology as described before and were left overnight. Then, small amounts of the gels were transferred to the cuvette for measurement.

**Fluorescence spectroscopy:** Data were collected on an Agilent Technologies Cary Eclipse fluorescence spectrometer. Samples were prepared in PMMA cuvettes with a path length of 1 cm by following the same procedure as mentioned before. All gels were left overnight before measurements were carried out. In all cases, the excitation wavelength was 300 nm. Both the excitation and emission slit widths were 5 nm.

**FTIR spectroscopy:** Data were recorded using an Agilent Cary 630 FTIR spectrometer (with ATR attachment). For the solid samples (amorphous), the background of the empty ATR crystal was taken. For gels, 20% DMSO-$d_6$ in $D_2O$ was used for the background correction. All the gels were prepared using DMSO-$d_6$, $D_2O$ and NaOD following the same methodology as described above. Then, small amounts of the gels were deposited on the ATR crystal before recording the spectra.

**Small Angle X-Ray Scattering (SAXS):** SAXS data were collected at beamline I22, Diamond Light Source. Sample were sealed into polycarbonate capillaries with an internal diameter of 1.8 mm, and SAXS data collected as a single frame, of 1 s duration, for each sample. Data were collected using an incident beam energy of 12.4 keV (wavelength 0.999 Å) and a sample to detector distance of 5.719 m. Sample to detector distance was calibrated using a standard sample of silver behenate.

2-Dimensional scattering images were corrected using the DAWN[2] software package, according to standard data reduction pipelines,[3] before being azimuthally integrated to give 1-dimensional scattering curves that were used in further analysis.

Scattering length densities (SLD) were calculated using the calculator available on the NIST website.[4]

The density of both gelators was estimated to be 1.55 g/mL for the purposes of this calculation.

Gelator **1**, $C_{30}H_{32}N_2O_5$: SLD = 14.025 x $10^{-6}$ Å$^{-2}$

Gelator **2**, $C_{17}H_{18}N_2O_2$: SLD = 13.907 x $10^{-6}$ Å$^{-2}$

For the mixture of **1** and **2**, the SLD was calculated from a weighted average of the SLD for each component: SLD = 13.95 x $10^{-6}$ Å$^{-2}$

Solvent mixture of DMSO/$H_2O$: SLD = 9.6024 x $10^{-6}$ Å$^{-2}$

The background solvent samples with and without enzyme are very similar, so just mixed solvent background was used for all data analysis. The fitting approach to each of the data sets is described below and the values from the fits are shown in Table S1 and Table S2. The data and the fits are shown in Figures S1-S4. All data were fitted using the Sasview software.[5]

The data for gelator **1** alone at low pH fits to a cylinder model combined with a power law to take into account the excess scattering at low Q. An arbitrary length of 500 nm was used and kept constant for the fit; allowing this parameter to vary did not result in other variables changing significantly but simply resulted in the length becoming longer that reasonable.

The data for gelator **2** at low pH fits to a power law only.

The data for the mixture of **1** and **2** at low pH fits to a cylinder model combined with a power law to take into account the excess scattering at low Q. Again, an arbitrary length of 500 nm was used and kept constant for the fit; allowing this parameter to vary did not result in other variables changing significantly but simply resulted in the length becoming longer that reasonable.

The data for **1** alone at high pH is of relatively low intensity and fits well to a power law.
**2** at high pH fits to a cylinder combined with a power law. A power law alone gives a chi squared of below 5 but misses the mid Q region. A cylinder alone requires a significant polydispersity and does not give a good fit to the data. Combining a power law and cylinder captures the data well.
The data for the mixture of **1** and **2** at high pH fits well to a flexible cylinder model, with an arbitrarily high length. It is possible to fit the data to a combined cylinder and power law, but the quality of the fit is not as good and the fit tends to iterate towards an extremely high power law scale, which results in a significantly worse fit to the data. On the basis of this, the fit to the flexible cylinder model was chosen as being more suitable.
The data for the mixture of **1** and **2** at high pH induced by the enzymatic reaction fits well to a flexible elliptical cylinder model. The fit to a flexible cylinder model is significantly worse.

| | **1** (low pH) | **2** (low pH) | **1** and **2** (low pH) |
|---|---|---|---|
| B/G | $7.84 \times 10^7$ | $7.52 \times 10^7$ | $5.77 \times 10^7$ |
| Scale (PL) | 5190 ± 79 | 217 ± 16 | 9075 ± 131 |
| PL | 3.71 ± 0.01 | 3.75 ± 0.01 | 3.41 ± 0.02 |
| Scale (C) | $9.1153 \times 10^6 \pm 5.98 \times 10^4$ | | $7.66 \times 10^6 \pm 23370$ |
| Radius (Å) | 35.4 ± 0.2 | | 44.8 ± 0.1 |
| Length (Å) | 5000* | | 5000* |
| Kuhn Length (Å) | | | |
| Axis radius | | | |
| Chi squared | 4.3097 | 1.0003 | 1.9194 |

**Table S1.** Fitting parameters for fits to SAXS data for **1**, **2** and mixtures of **1** and **2** at low pH.

| | **1** (high pH) | **2** (high pH) | **1** and **2** (high pH) | **1** and **2** (high pH; enzyme) |
|---|---|---|---|---|
| B/G | $4.23 \times 10^7$ | $6.72 \times 10^7$ | $2.91 \times 10^7$ | $4.72 \times 10^7$ |
| Scale (PL) | 5502 ± 584 | 2.46 ± 0.19 | | |
| PL | 2.92 ± 0.02 | 4.67 ± 0.01 | | |
| Scale (C) | | $4.81 \times 10^5 \pm 1.39 \times 10^4$ | $2.91 \times 10^7 \pm 36055$ | $2.27 \times 10^7 \pm 8.52 \times 10^4$ |
| Radius (Å) | | 53.1 ± 1.2 | 38.4 ± 0.04 | 33.4 ± 0.07 |
| Length (Å) | | 5000* | 5000* | 4400 ± 390 |
| Kuhn Length (Å) | | | 231 ± 1 | 592 ± 9 |
| Axis radius | | | | 1.95 ± 0.01 |
| Chi squared | 0.84203 | 1.1209 | 7.6251 | 2.9694 |

**Table S2.** Fitting parameters for fits to SAXS data for **1**, **2** and mixtures of **1** and **2** at high pH.

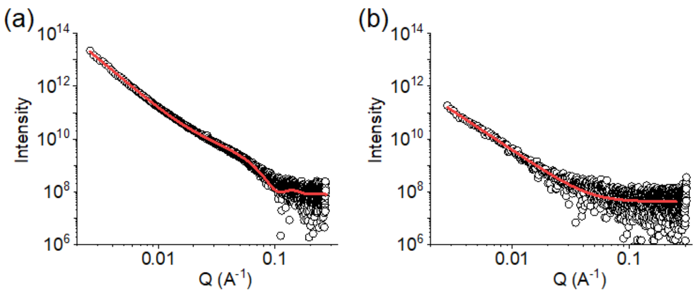


**Figure S1.** SAXS data and fits for **1** at (a) low pH and (b) high pH. The circles show the data and the red lines the fits to the data.

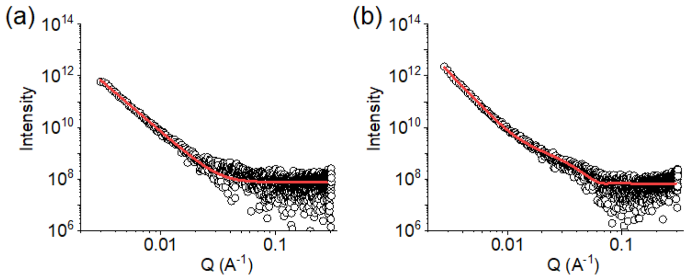


**Figure S2.** SAXS data and fits for **2** at (a) low pH and (b) high pH. The circles show the data and the red lines the fits to the data.

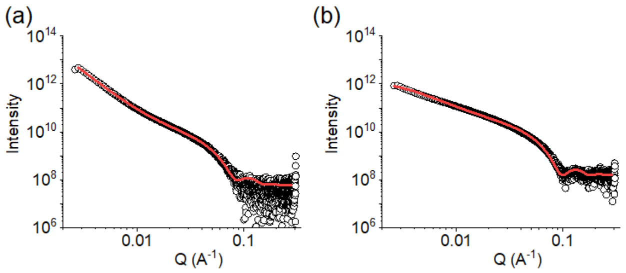


**Figure S3.** SAXS data and fits for the mixture of **1** and **2** at (a) low pH and (b) high pH. The circles show the data and the red lines the fits to the data.

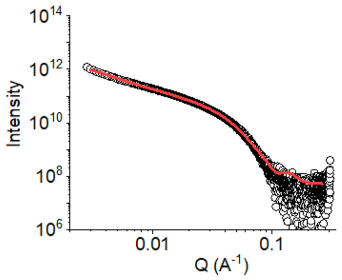


**Figure S4.** SAXS data and fits for the mixture of **1** and **2** at high pH obtained from the enzymatic reaction involving initial conditions: initial concentrations of **1** and **2** are 2 mg/mL, [urease] = 0.5 mg/mL, [urea] = 0.1 M, solvent is DMSO/$H_2O$ (20/80, v/v). The circles show the data and the red line the fits to the data.

**Supplementary Figures**

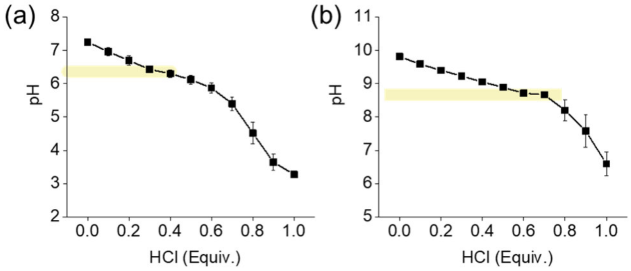


**Figure S5**. (a, b) Determination of apparent p$K_a$ of (a) **1** (2 mg/mL) and (b) **2** (2 mg/mL) in 20:80 DMSO/water (v/v). The plateau is taken to represent the apparent p$K_a$ value, shown by the horizontal shading.

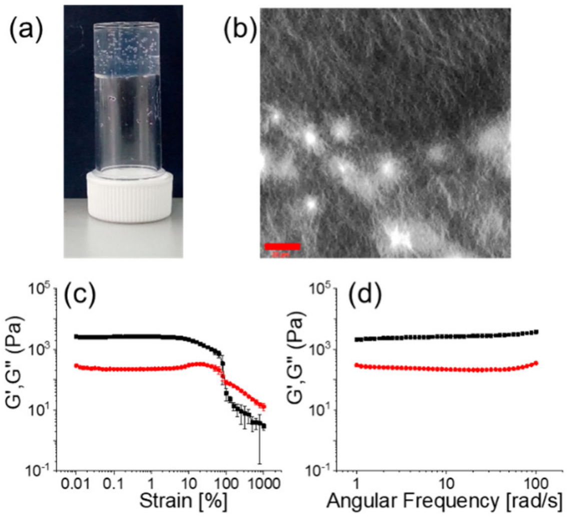


**Figure S6**. (a) Photograph, (b) confocal fluorescence microscopy image (scale bar represents 20 μm), (c) strain and (d) frequency sweep experiments of the hydrogel of **1**. The structures visible in the gel are air bubbles, not precipitation. For (c) and (d), the black data represent G′ and the red data G″. In all cases, concentration of **1** is 2 mg/mL and solvent is 20:80 DMSO/water (v/v).

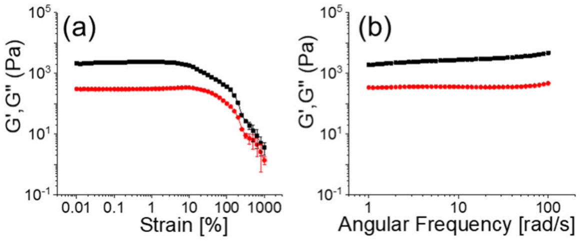


**Figure S7**. (a) Strain and (b) frequency sweep experiments of the multicomponent gel of **1** and **2** obtained from DMSO/water (20:80, v/v). In both cases, initial concentrations of **1** and **2** are 2 mg/mL. The black data represent G′ and the red data G″.

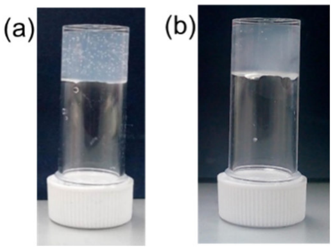


**Figure S8**. Photographs of the multicomponent gel of **1** and **2** obtained in absence (a) and presence (b) of NaOH. In both cases, initial concentrations of **1** and **2** are 2 mg/mL, concentration of NaOH is 0.01 M, solvent is 20:80 DMSO/water (v/v).

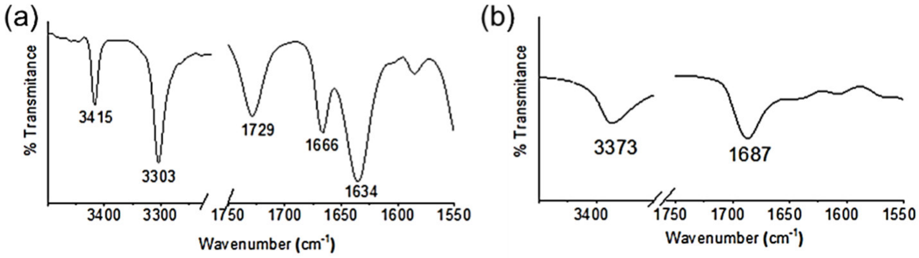


**Figure S9**. Partial FTIR spectra of (a) **1** and (b) **2** in their amorphous (solid) states.

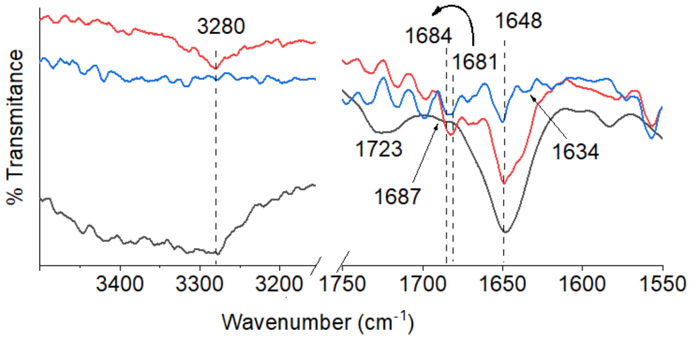


**Figure S10**. Partial FTIR spectra of the hydrogel of **1** (black) and the multicomponent gels of **1** and **2** obtained at pH 3.3 (red) and 10.2 (blue).

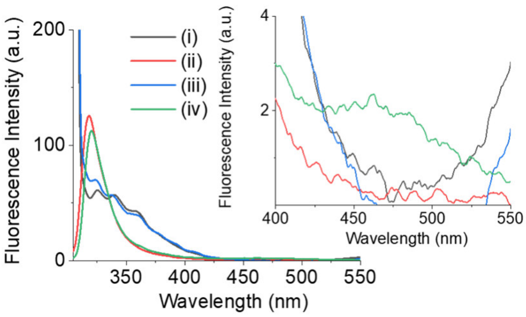


**Figure S11**. Emission spectra of **1** (i, iii) and **2** (ii, iv) in DMSO/water (20/80, v/v) in absence (i, ii) and presence (iii, iv) of equimolar amounts of NaOH. Inset shows an expanded section of the graph. In all cases, initial concentrations of **1** and **2** are 2 mg/mL.

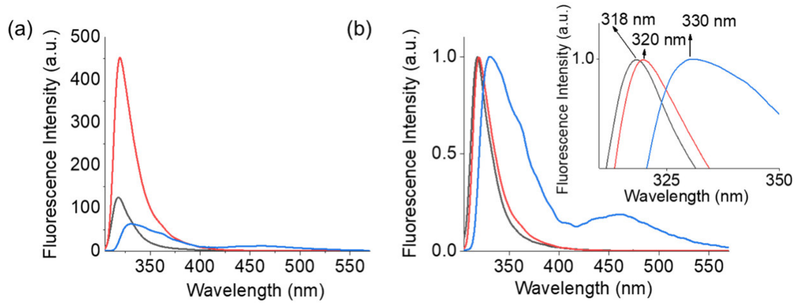

**Figure S12**. (a) Emission spectra of a solution of **2** (black) and the multicomponent gels of **1** and **2** obtained in absence (red) and presence (blue) of NaOH. Figure (b) is the normalized graph of (a). Inset shows an expanded section of the graph. In all cases, initial concentrations of **1** and **2** are 2 mg/mL, concentration of NaOH is 0.01 M, solvent is 20:80 DMSO/water (v/v).

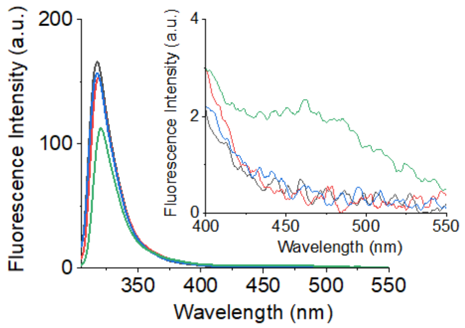

**Figure S13**. (a) Emission spectra of **2** in DMSO/water (20/80, v/v) in absence (black, red) and presence (blue, green) of equimolar amounts of NaOH. Initial concentration of **2** is 1 mg/mL (black and blue) and 2 mg/mL (red and green). Inset shows an expanded section of the graph.

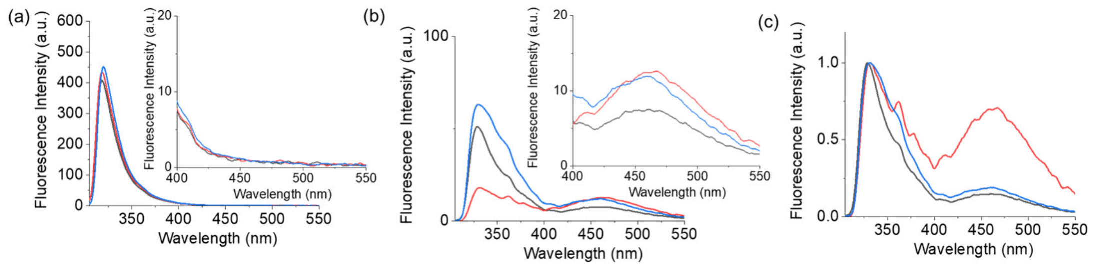

**Figure S14**. Emission spectra of the mixtures of **1** and **2** in DMSO/water (20/80, v/v) in (a) absence and (b) presence of NaOH. In both cases, initial conditions: (black) [**1**] = [**2**] = 1 mg/mL, (red) [**1**] = 1 mg/mL, [**2**] = 2 mg/mL, (blue) [**1**] = 2 mg/mL, [**2**] = 2 mg/mL. For (b), in all cases, concentration of NaOH is 0.01 M. For (a) and (b), Insets show an expanded section of the graphs. (Figure (c) represents the normalized graph of (b).

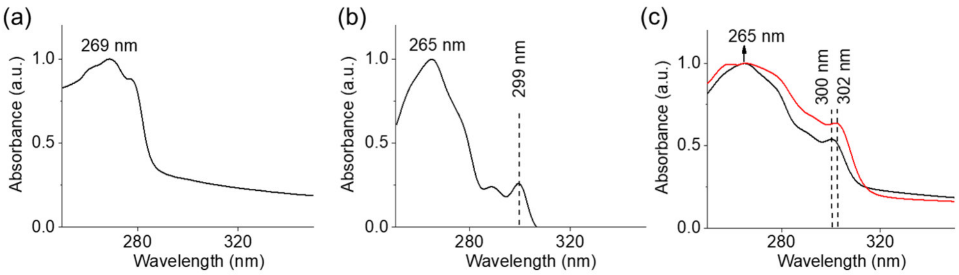


**Figure S15**. Normalized UV-vis spectra of (a) hydrogel of **1**, (b) solution of **2**, and (c) the multicomponent gels of **1** and **2** obtained in absence (black) and presence (red) of NaOH. In all cases, initial concentrations of **1** and **2** are 2 mg/mL, concentration of NaOH is 0.01 M, solvent is 20:80 DMSO/water (v/v).

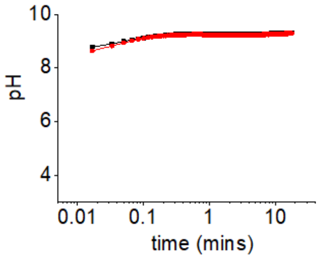


**Figure S16**. Variation of pH with time for the urea-urease reaction in water (black) and 20/80 DMSO/water (v/v) (red) in absence of both **1** and **2**. In both cases, initial [urease] = 0.5 mg/mL, [urea] = 0.1 M.

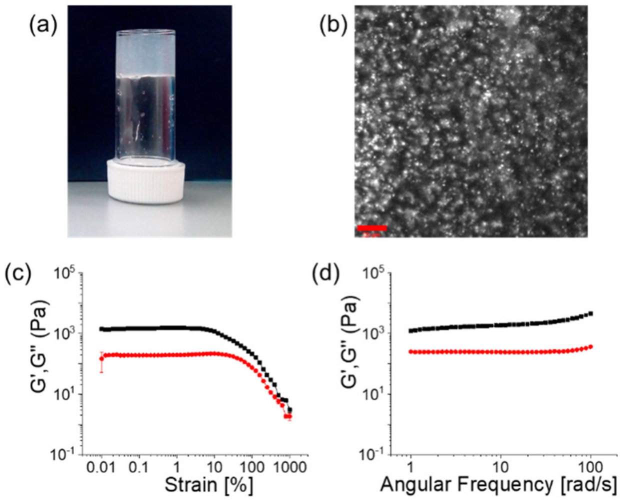


**Figure S17**. (a) Photograph, (b) confocal fluorescence microscopy image (scale bar represents 20 μm), (c) strain and (d) frequency sweep experiments of the multicomponent gel of **1** and **2** obtained in presence of urease. For (c, d), the black data represent G′ and the red data G″. In all cases, initial concentrations of **1** and **2** are 2 mg/mL, [urease] = 0.5 mg/mL, solvent is 20:80 DMSO/water (v/v).

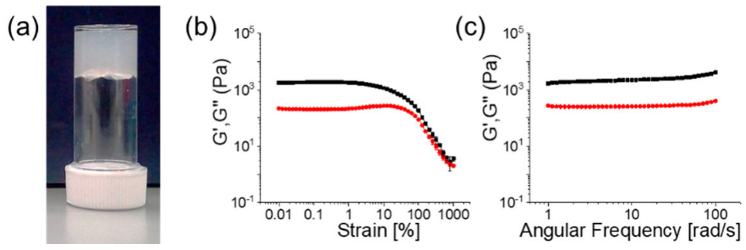


**Figure S18**. (a) Photograph, (b) strain and (c) frequency sweep experiments of the multicomponent gel of **1** and **2** obtained from the enzymatic reaction involving initial conditions: concentrations of **1** and **2** are 2 mg/mL, [urease] = 0.5 mg/mL, [urea] = 0.1 M. Solvent is 20:80 DMSO/water (v/v). For (b, c), the black data represents G′ and the red data G″.

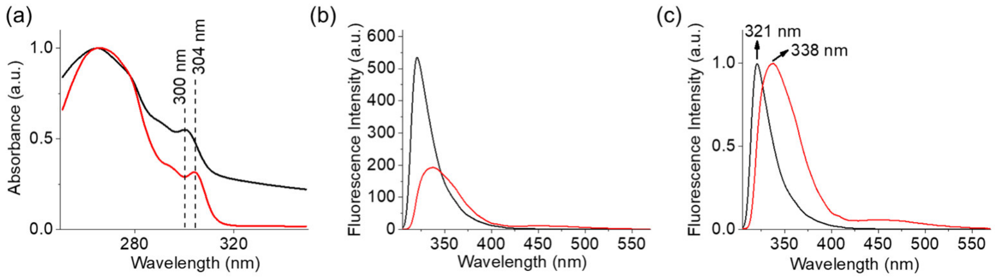


**Figure S19**. (a) Normalized UV-vis and (b) emission spectra of the multicomponent gels of **1** and **2** obtained from DMSO/water (20:80, v/v) involving initial conditions: (black) [urease] = 0.5 mg/mL, [urea] = 0 M; (red) [urease] = 0.5 mg/mL, [urea] = 0.1 M. In all cases, initial concentrations of **1** and **2** are 2 mg/mL. Figure (c) is the normalized graph of (b).

| Conditions (solvent is 20% DMSO in water) | [**1**] in mg/mL | [**2**] in mg/mL | Final pH | From frequency sweep [at 10 rad/s] | | Critical strain (%) | %Strain at crossover point |
|---|---|---|---|---|---|---|---|
| | | | | G′ (Pa) | G″ (Pa) | | |
| 0.1 M of NaOH | 2 | 2 | 10.2 | 685 | 80 | 13 | 48 |
| Enzymatic reaction with [urease] = 0.5 mg/mL, [urea] = 0.1 M | 2 | 2 | 9.2 | 2250 | 270 | 3 | - |

**Table S3.** Comparison of rheological data of the multicomponent gels of **1** and **2** prepared by different methods.

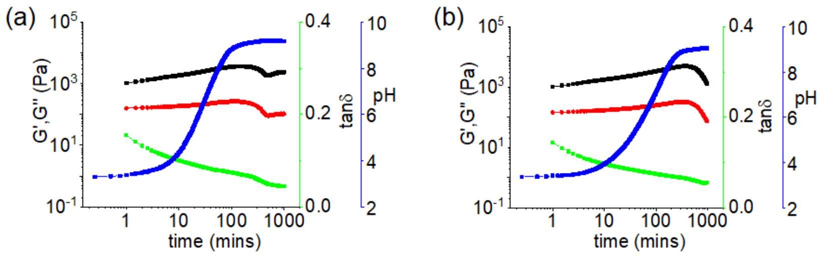


**Figure S20**. Variation of pH (blue), G′ (black), G″ (red) and tanδ (green) with time for the mixture of **1** and **2** in presence of urea-urease reaction involving initial conditions: (a) [urease] = 0.5 mg/mL, [urea] = 0.06 M; (b) [urease] = 0.3 mg/mL, [urea] = 0.1 M. In both cases, initial concentrations of **1** and **2** are 2 mg/mL and solvent is 20:80 DMSO/water (v/v).

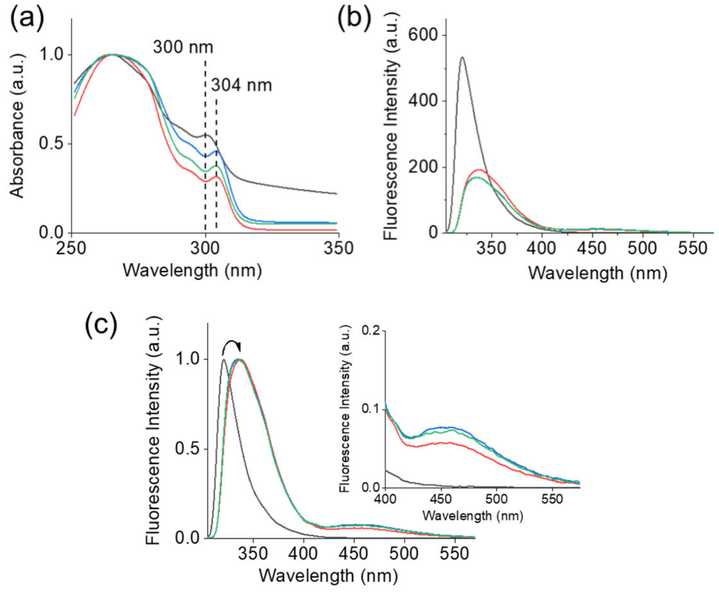


**Figure S21**. (a) Normalized UV-vis and (b) emission spectra of the multicomponent gels of **1** and **2** obtained from DMSO/water (20:80, v/v) involving initial conditions: (black) [urease] = 0.5 mg/mL, [urea] = 0 M; (red) [urease] = 0.5 mg/mL, [urea] = 0.1 M; (blue) [urease] = 0.5 mg/mL, [urea] = 0.06 M; (green) [urease] = 0.3 mg/mL, [urea] = 0.1 M. In all cases, initial concentrations of **1** and **2** are 2 mg/mL. Figure (c) is the normalized graph of (b). Inset represents an expanded section of the graph.

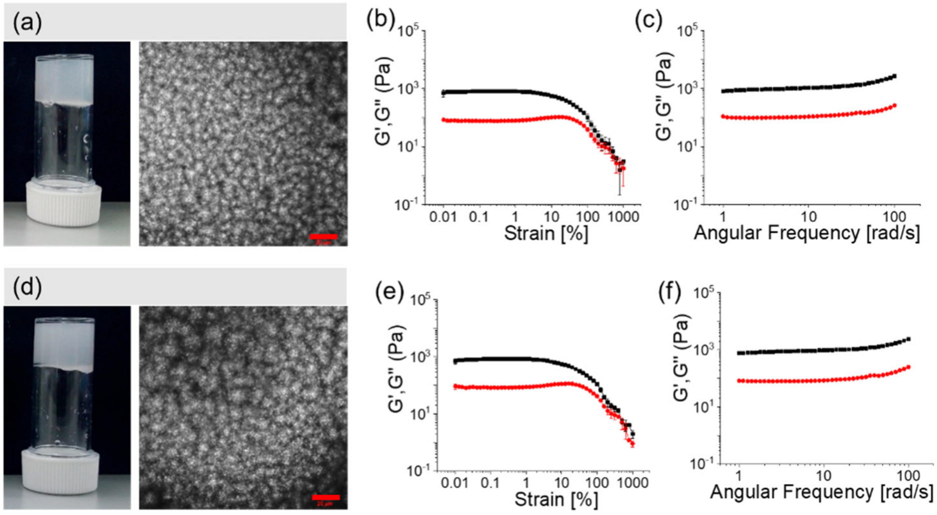


**Figure S22**. (a, d) Confocal microscopy images (scale bar is 20 μm), (b, e) strain and (c, f) frequency sweep experiments of the multicomponent gel of **1** and **2** obtained from the enzymatic reaction involving initial conditions: (a-c) [urease] = 0.5 mg/mL, [urea] = 0.06 M; (d-f) [urease] = 0.3 mg/mL, [urea] = 0.1 M. In all cases, initial concentrations of **1** and **2** are 2 mg/mL and solvent is 20:80 DMSO/water (v/v). For (b, c, e, f), the black data represent G′ and the red data G″.

| Conditions (solvent is 20% DMSO in water) | [urease] in mg/mL | [urea] in M | Final pH | From frequency sweep [at 10 rad/s] | | Critical strain (%) | %Strain at crossover point |
|---|---|---|---|---|---|---|---|
| | | | | G′ (Pa) | G″ (Pa) | | |
| [**1**] = [**2**] = 2 mg/mL | 0.5 | 0.1 | 9.2 | 2250 | 270 | 3 | - |
| | 0.5 | 0.06 | 9.2 | 1055 | 110 | 4 | 695 |
| | 0.3 | 0.1 | 9.1 | 960 | 85 | 4 | - |

**Table S4.** Comparison of rheological data of the multicomponent gels of **1** and **2** prepared by different methods.

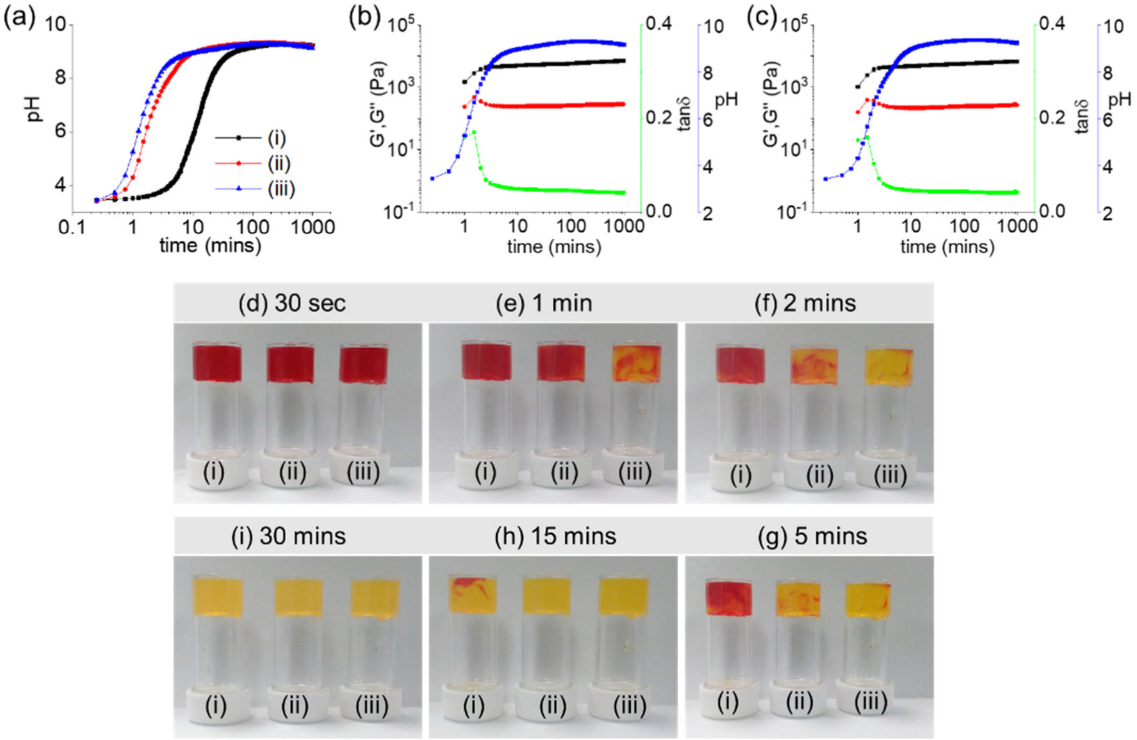


**Figure S23.** (a) Variation of pH with time for the multicomponent gel of **1** and **2** involving urea-urease reaction in absence (i) and presence of 0.002 M (ii) and 0.006 M of $CaCl_2$. In all cases, initial concentrations of **1** and **2** are 2 mg/mL, [urease] = 0.5 mg/mL, [urea] = 0.1 M and solvent is DMSO/$H_2O$ (20:80, v/v). (b) and (c) represent the variation of pH (blue), G′ (black), G″ (red) and tanδ (green) with time for the mixture of **1** and **2** in presence of urea-urease reaction involving initial conditions (ii) and (iii) respectively. (d-i) Associated color change of the gels with time involving experiment (a). For (d-i), methyl red (0.05 mg/mL) is used to dye the gels.

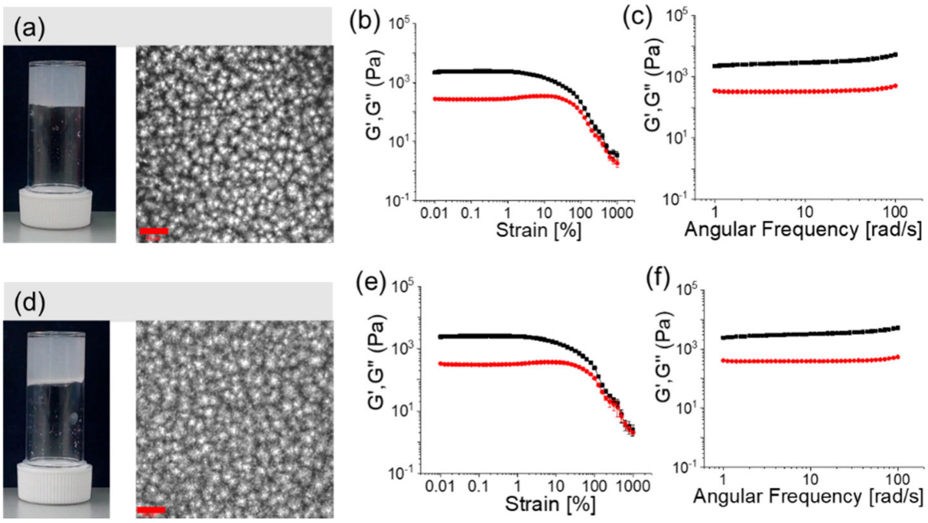


**Figure S24**. (a, d) Confocal microscopy images (scale bar is 20 μm), (b, e) strain and (c, f) frequency sweep experiments of the multicomponent gel of **1** and **2** obtained from the enzymatic reaction involving 0.002 M (a-c) and 0.006 M (d-f) of $CaCl_2$. In all cases, initial concentrations of **1** and **2** are 2 mg/mL, [urease] = 0.5 mg/mL, [urea] = 0.1 M and solvent is DMSO/$H_2O$ (20:80, v/v). For (b, c, e, f), the black data represent G′ and the red data G″.

| Conditions (solvent is 20% DMSO in water) | $[CaCl_2]$ in M | Final pH | From frequency sweep [at 10 rad/s] | | Critical strain (%) | %Strain at crossover point |
|---|---|---|---|---|---|---|
| | | | G′ (Pa) | G″ (Pa) | | |
| [**1**] = [**2**] = 2 mg/mL<br>[urease] = 0.5 mg/mL<br>[urea] = 0.1 M | 0 | 9.2 | 2250 | 270 | 3 | - |
| | 0.002 | 9.2 | 2890 | 330 | 4 | - |
| | 0.006 | 9.2 | 3175 | 385 | 4 | 595 |

**Table S5.** Comparison of rheological data of the multicomponent gels of **1** and **2** prepared by different methods.

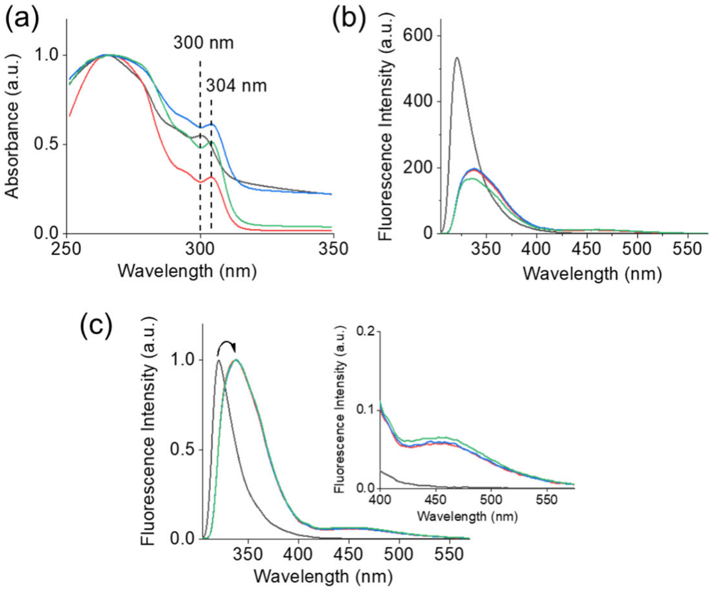


**Figure S25**. (a) Normalized UV-vis and (b) emission spectra of the multicomponent gels of **1** and **2** obtained from the enzymatic reaction involving initial conditions: (black) [urease] = 0.5 mg/mL, [urea] = 0 M, $[CaCl_2]$ = 0 M; (red) [urease] = 0.5 mg/mL, [urea] = 0.1 M, $[CaCl_2]$ = 0 M; (blue) [urease] = 0.5 mg/mL, [urea] = 0.1 M, $[CaCl_2]$ = 0.002 M; (green) [urease] = 0.5 mg/mL, [urea] = 0.1 M, $[CaCl_2]$ = 0.006 M. In all cases, initial concentrations of **1** and **2** are 2 mg/mL and solvent is DMSO/$H_2O$ (20:80, v/v). Figure (c) is the normalized graph of (b). Inset represents an expanded section of the graph.

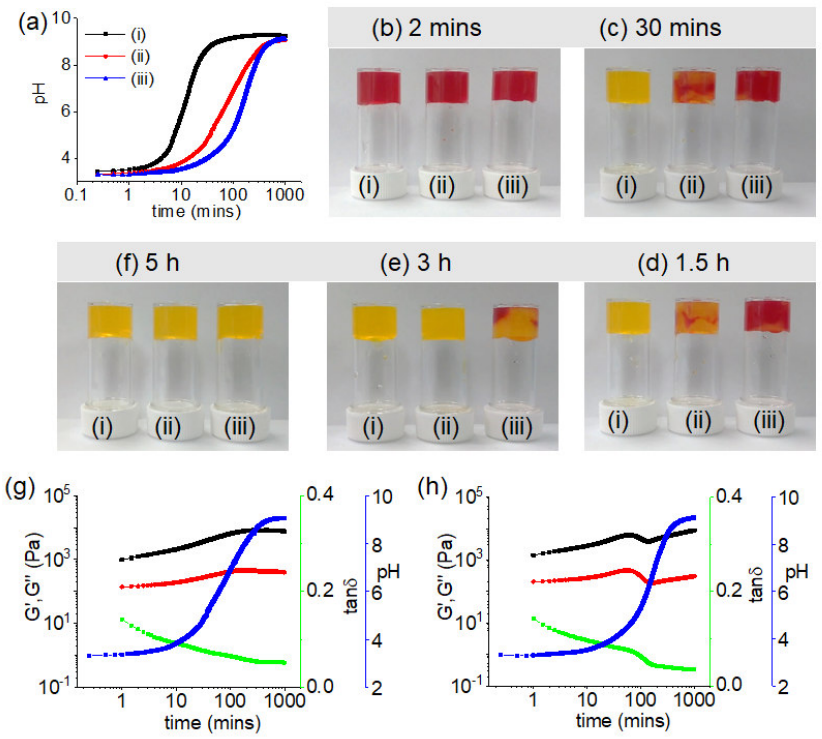


**Figure S26**. (a) Variation of pH with time along with associated color change (b-f) of the corresponding hydrogels of (**1**+**2**) in presence of urea-urease reaction involving initial conditions: (i) [**1**] = 2 mg/mL, [**2**] = 2 mg/mL; (ii) [**1**] = 2 mg/mL, [**2**] = 3 mg/mL; (iii) [**1**] = 3 mg/mL, [**2**] = 2 mg/mL. In all cases, initial [urease] = 0.3 mg/mL, [urea] = 0.1 M and solvent is DMSO/$H_2O$ (20:80, v/v). (g) and (h) represent the variation of pH (blue), G′ (black), G″ (red) and tanδ (green) with time for the mixture of **1** and **2** in presence of urea-urease reaction involving initial conditions (ii) and (iii) respectively. For (b-f), methyl red (0.05 mg/mL) is used to dye the gels.

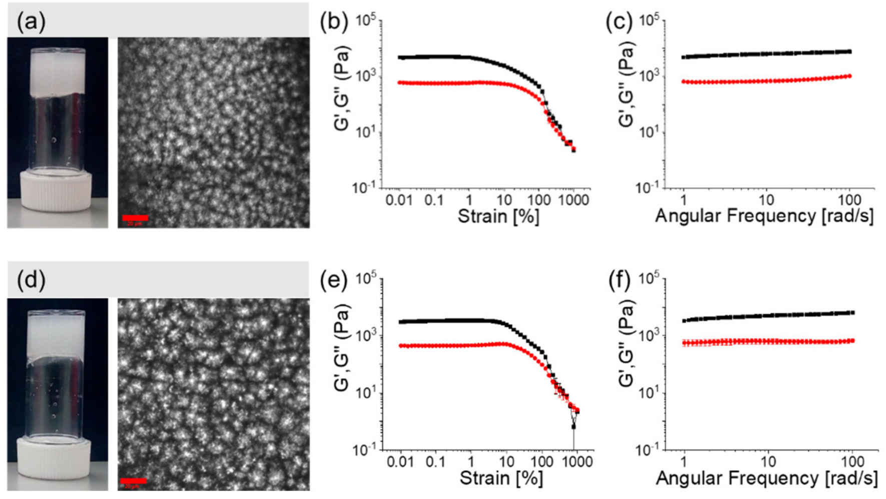


**Figure S27**. (a, d) Confocal microscopy images (scale bar is 20 μm), (b, e) strain and (c, f) frequency sweep experiments of the multicomponent gel of **1** and **2** obtained from the enzymatic reaction involving initial conditions: (a-c) [**1**] = 2 mg/mL, [**2**] = 3 mg/mL; (d-f) [**1**] = 3 mg/mL, [**2**] = 2 mg/mL. In all cases, initial [urease] = 0.3 mg/mL, [urea] = 0.1 M and solvent is DMSO/$H_2O$ (20:80, v/v). For (b, c, e, f), the black data represent G′ and the red data G″.

| Conditions (solvent is 20% DMSO in water) | [**1**] in mg/mL | [**2**] in mg/mL | Final pH | From frequency sweep [at 10 rad/s] | | Critical strain (%) | %Strain at crossover point |
|---|---|---|---|---|---|---|---|
| | | | | G′ (Pa) | G″ (Pa) | | |
| [urease] = 0.5 mg/mL [urea] = 0.1 M | 2 | 2 | 9.2 | 2250 | 670 | 3 | - |
| | 2 | 3 | 9.2 | 6305 | 635 | 3 | 485 |
| | 3 | 2 | 9.1 | 4905 | 85 | 8 | 560 |

**Table S6.** Comparison of rheological data of the multicomponent gels of **1** and **2** prepared by different methods.

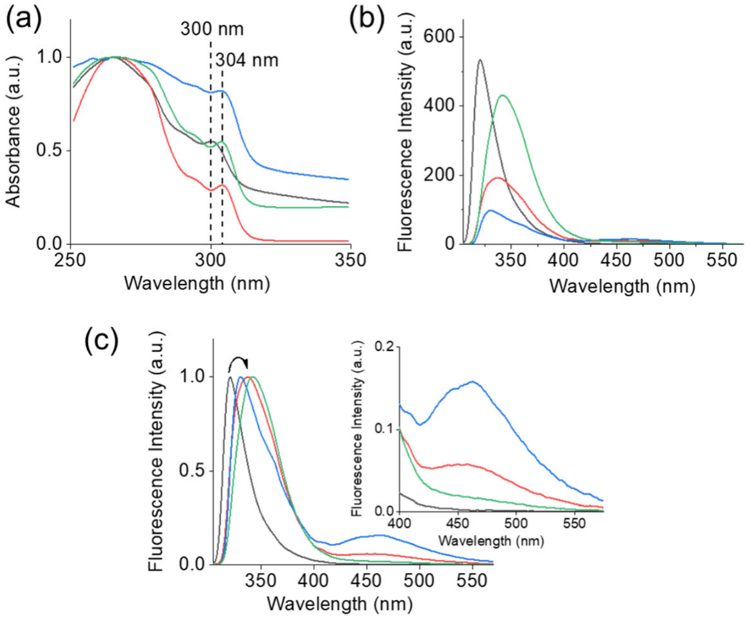


**Figure S28**. (a) Normalized UV-vis and (b) emission spectra of the multicomponent gels of **1** and **2** obtained from the enzymatic reaction involving initial conditions: (black) [**1**] = 2 mg/mL, [**2**] = 2 mg/mL, [urease] = 0.5 mg/mL, [urea] = 0 M; (red) [**1**] = 2 mg/mL, [**2**] = 2 mg/mL, [urease] = 0.5 mg/mL, [urea] = 0.1 M; (blue) [**1**] = 2 mg/mL, [**2**] = 3 mg/mL, [urease] = 0.5 mg/mL, [urea] = 0.1 M; (green) [**1**] = 3 mg/mL, [**2**] = 2 mg/mL, [urease] = 0.5 mg/mL, [urea] = 0.1 M. In all cases, solvent is DMSO/$H_2O$ (20:80, v/v). Figure (c) is the normalized graph of (b). Inset represents an expanded section of the graph.

**References**


(1) Nolan, M. C.; Fuentes Caparrós, A. M.; Dietrich, B.; Barrow, M.; Cross, E. R.; Bleuel, M.; King, S. M.; Adams, D. J. *Soft Matter* **2017**, *13*, 8426.

(2) Filik, J.; Ashton, A. W.; Chang, P. C. Y.; Chater, P. A.; Day, S. J.; Drakopoulos, M.; Gerring, M. W.; Hart, M. L.; Magdysyuk, O. V.; Michalik, S.; Smith, A.; Tang, C. C.; Terrill, N. J.; Wharmby, M. T.; Wilhelm, H. *J. Appl. Cryst.,* **2017**, *50*, 959.

(3) Pauw, B. R.; Smith, A. J.; Snow, T.; Terrill, N. J.; Thunemann, A. F. *J. Appl. Cryst.* **2017**, *50*, 1800.

(4) https://www.ncnr.nist.gov/resources/activation/.

(5) http://www.sasview.org/.
